# Little evidence for vibrissal capsular muscles in humans

**DOI:** 10.1007/s12565-024-00757-7

**Published:** 2024-02-03

**Authors:** Sven Schumann

**Affiliations:** Institute of Anatomy, University Medical Center of the Johannes Gutenberg‐University Mainz, Johann-Joachim-Becher-Weg 13, 55128 Mainz, Germany

Dear Professor Takeda,

With great interest, I have read the review about atavistic and vestigial anatomical structures in the human body by Dhawan (Dhawan et al. 2023). I want to congratulate the authors for this sophisticated publication but also share my personal opinion about one specific topic mentioned in the review.

Vibrissae (pili tactiles, whiskers, sinus hairs) are specialised sensory hairs which respond to mechanical stimuli. Vibrissae are characterised by a specific morphology. The thickened hairs are emitted from the follicle sinus complex (FSC) which contains the hair follicle surrounded by two large blood sinuses (the upper ring sinus and the lower cavernous sinus). The FSC is covered by a thick collagenous capsule and a layer of skeletal muscle. The musculature derives from the facial muscles which move the upper lip and the wing of the nose. The FSC is densely innervated. Sensory information from vibrissae is transmitted mainly via the infraorbital nerve (from the maxillary branch of the trigeminal nerve).

**Fig. 1:**
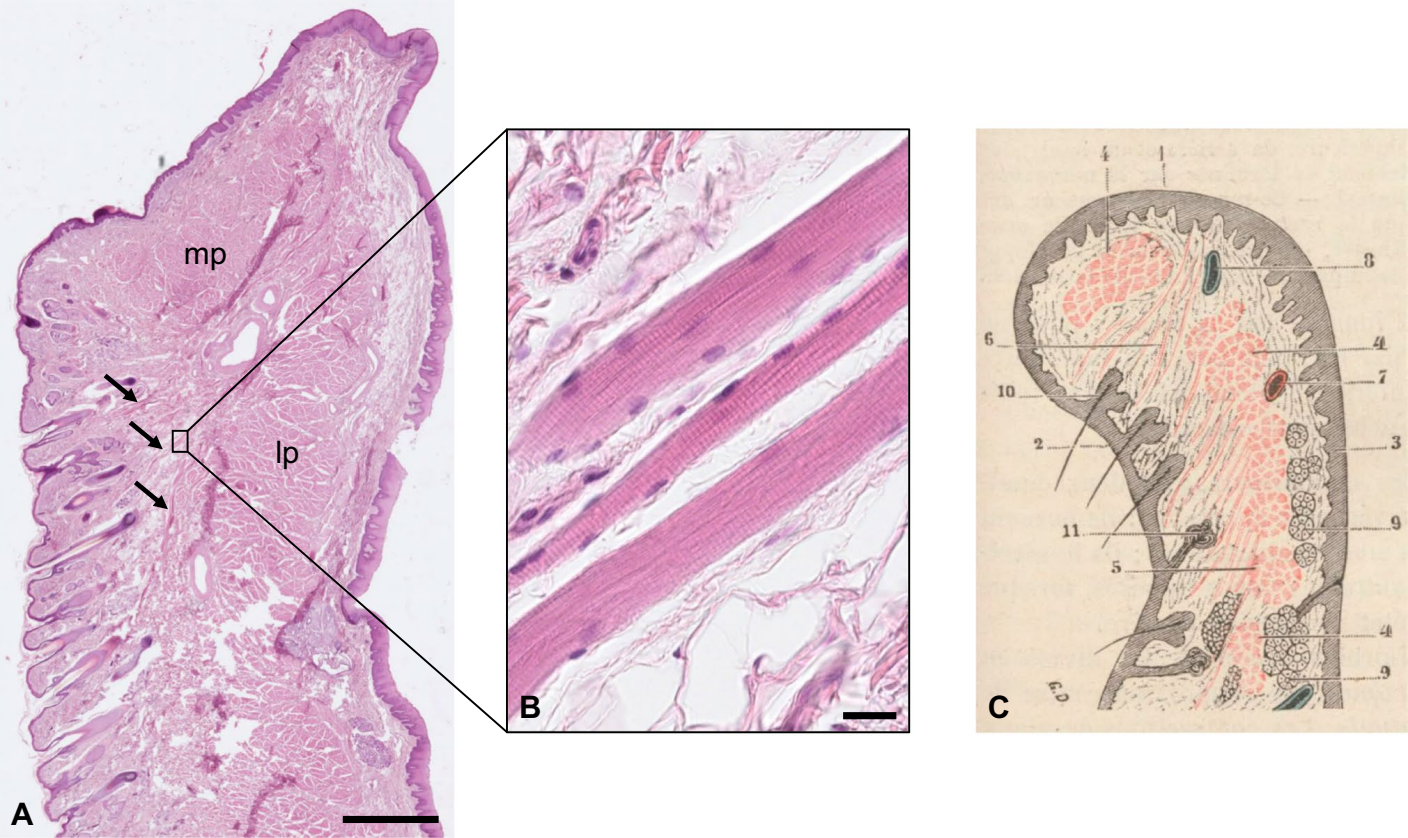
**A**: Human lower lip. Marginal part (mp) and labial part (lp) of the orbicularis oris muscle are indicated. Bundles of skeletal muscle fibres are visible (arrows). Bar = 2000 μm, hematoxylin and eosin stain, specimen from the histological teaching collection Mainz, Germany. **B**: Skeletal muscle fibre bundle in higher magnification. Bar = 20 μm. **C**: Schematic drawing of the inferior lip (taken from Testut 1899, Fig. 602). The muscle compresseur des lèvres de KLEIN is marked with 6

As mentioned in the review, Tamatsu postulated the existence of vestiges of vibrissal capsular muscles in the human upper lip (Tamatsu et al. 2007) of Japanese females and males. Unfortunately, there are several ambiguities concerning this study (e.g. confusion of blood vessels with hair follicles). Similar bundles of striated muscle fibres are visible not only in the human upper lip, but also in the lower lip (Fig. 1 A + B). These fibre bundles more likely represent the rectus labii muscle, which passes from the oral mucosa to the skin (synonyms: compressor labii muscle (Klein 1868); muscle compresseur des lèvres de KLEIN (Testut 1899)) (Fig. 1 C). In addition, I never observe blood sinuses or similar structures surrounding human face hairs in West-European body donors.

To my knowledge, there are no more publications supporting the existence of vibrissal capsular muscle residues in humans. Therefore, I only see little evidence for the existence of these structures. I want to encourage all anatomists to share own findings about this topic, especially since there is a controversy about the presence (Narisawa and Kohda 1993) or absence (Standring 2016) of arrector pili muscles in the face and the possibility of different findings in different populations.

Kind regards,

Sven Schumann.
